# Supplementary material for: Structure of the dopamine D2 receptor in complex with the antipsychotic drug spiperone
Source: Nat Commun. 2020 Dec 22;11:6442. doi: 10.1038/s41467-020-20221-0 (PMC7755896; doi:10.1038/s41467-020-20221-0)
Supplement: Supplementary file 1 — Supplementary Information [file 41467_2020_20221_MOESM1_ESM.pdf]

Supplementary Information

Structure of the dopamine D_2 receptor in complex with the antipsychotic drug spiperone

D. Im *et al*.

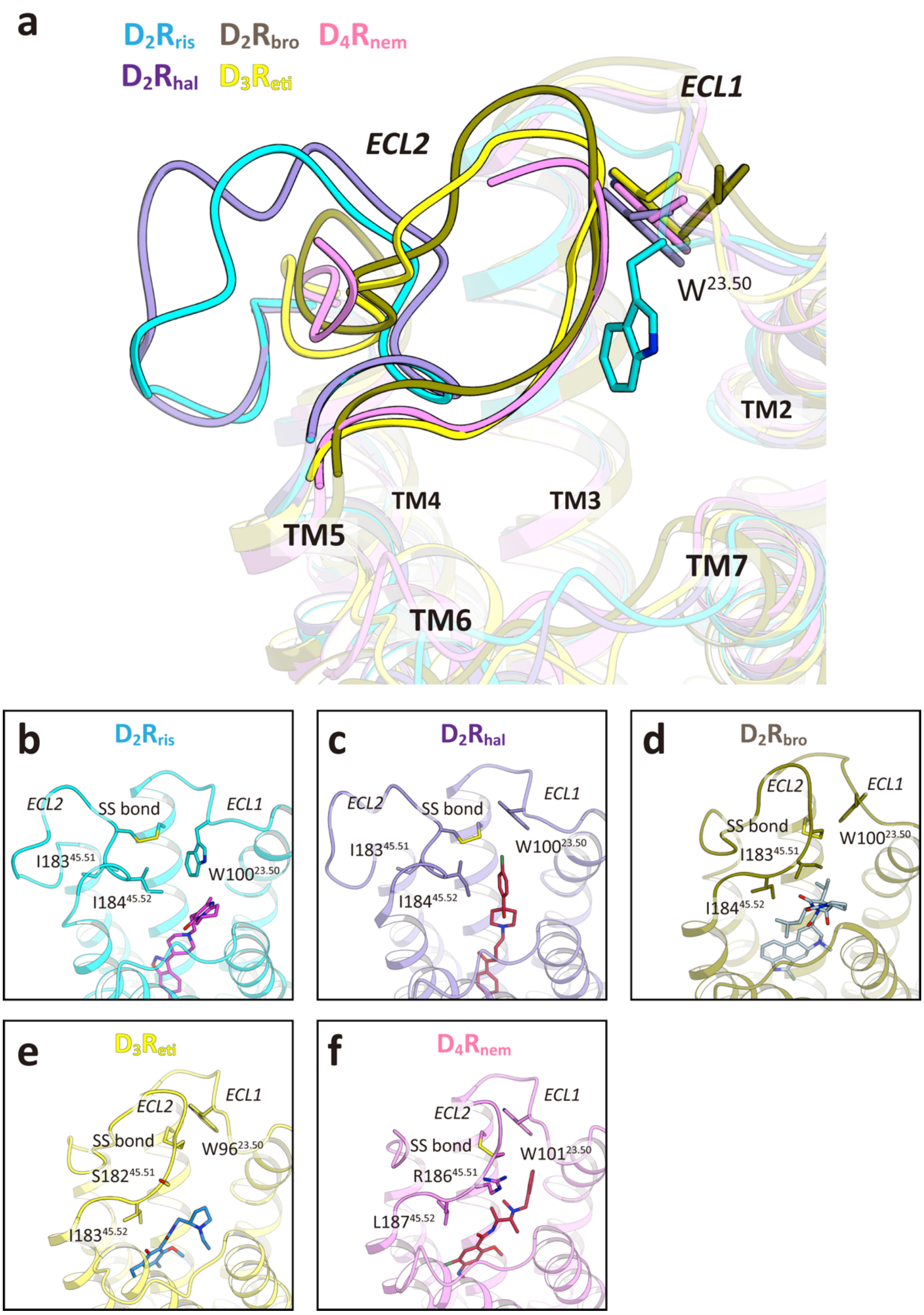

Supplementary Fig. 1. Comparison of the ECL2 conformation of D_2-class receptors. a, Extracellular view of superposition of D_2R_{ris}, D_2R_{hal}, D_2R_{bro}, D_3R_{eti} and D_4R_{nem} around ECL2. The conformation of ECL2 of D_2R_{ris} (**b**), D_2R_{hal} (**c**), D_2R_{bro} (**d**), D_3R_{eti} (**e**) and D_4R_{nem} (**f**). D_2R_{ris} (cyan), D_2R_{hal} (purple), D_2R_{bro} (olive), D_3R_{eti} (yellow), D_4R_{nem} (pink), risperidone (magenta), haloperidol (ivory), bromocriptine (lightblue), eticlopride (blue), and nemonapride (red) are shown.

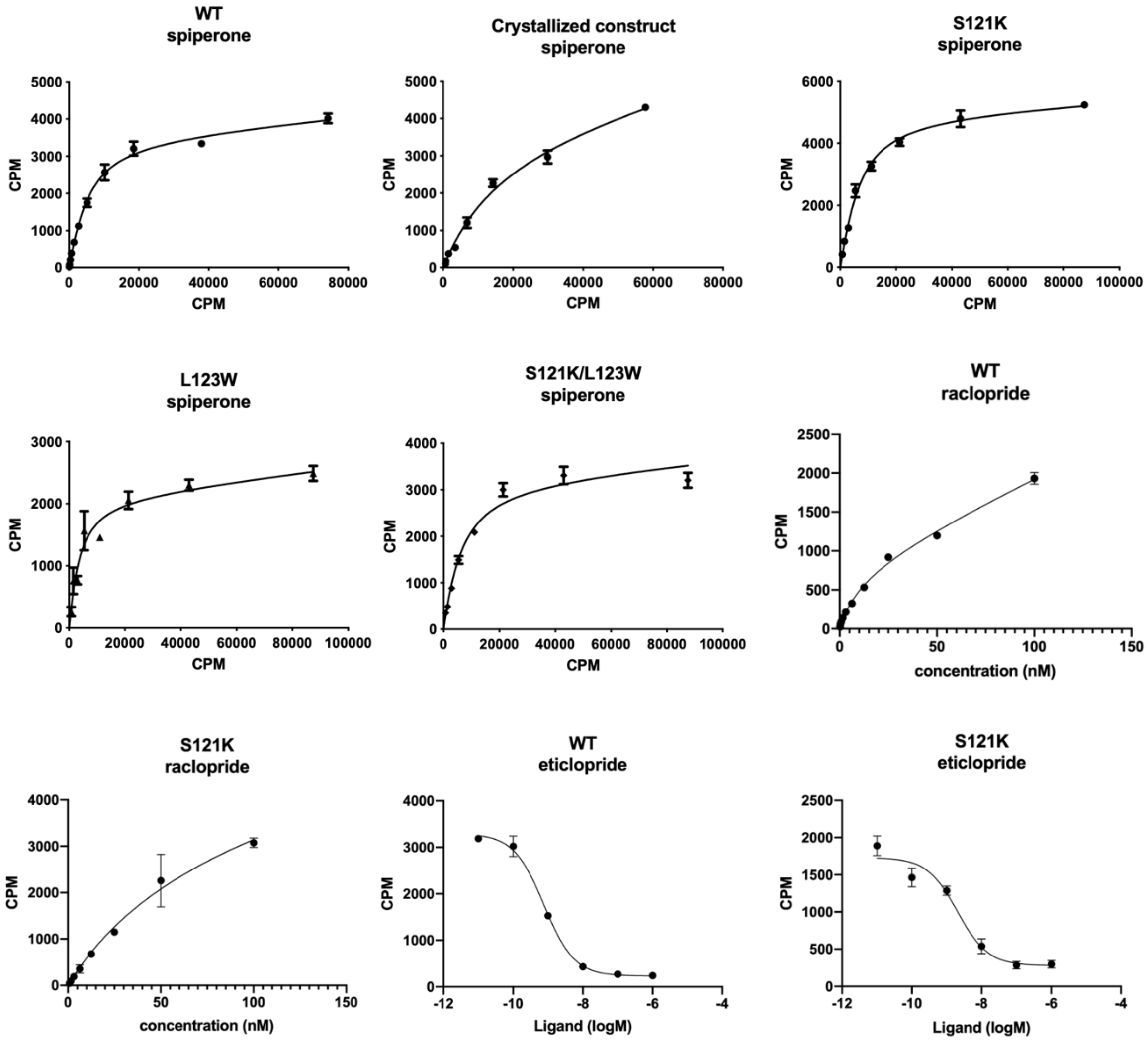

Supplementary Fig. 2. The displacement curves of the wild type (WT) and the mutants D_2Rs. The detailed values are shown in Supplementary Table 2. Data represent mean ± SEM from 3 biologically independent experiments.

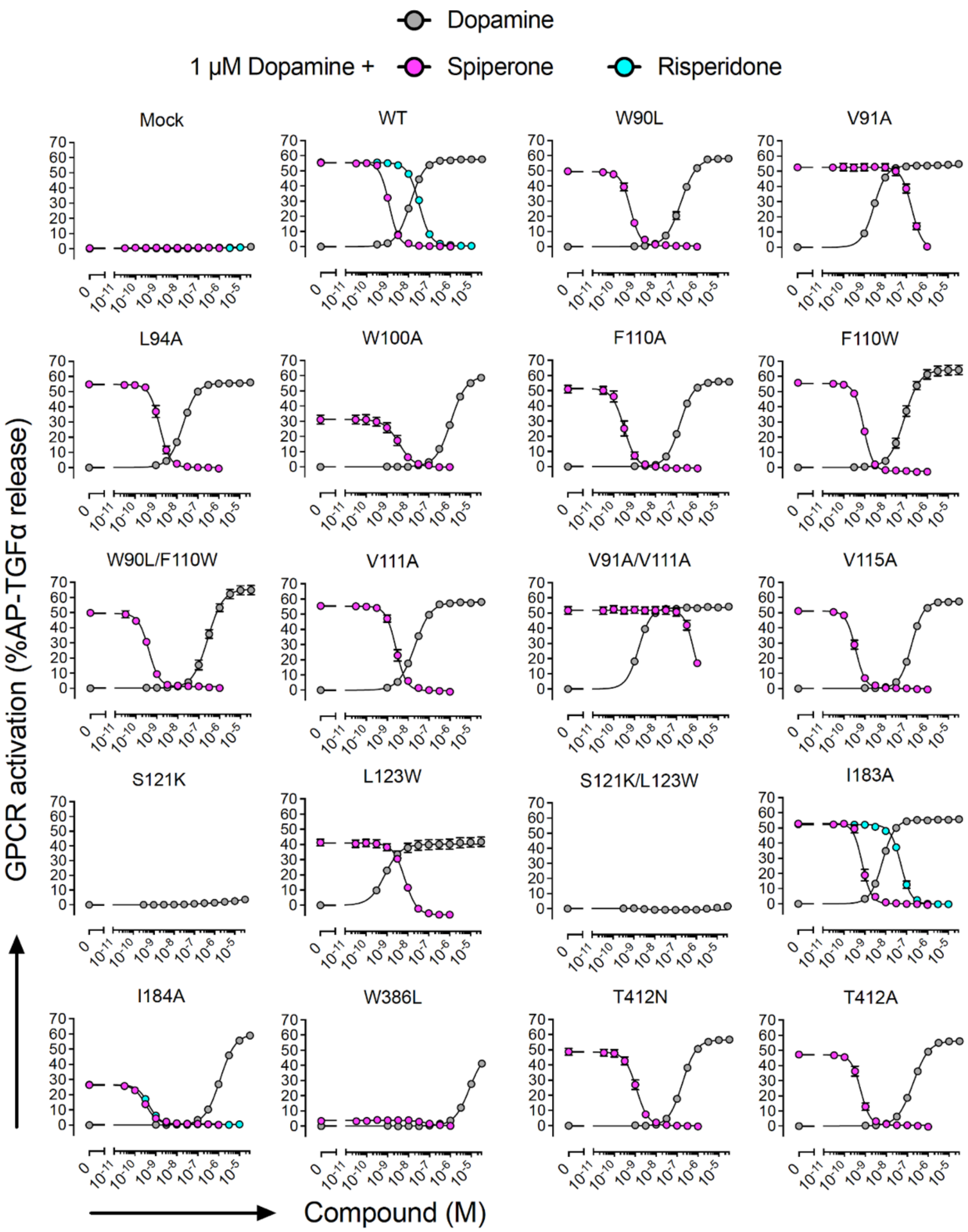

Supplementary Fig. 3. TGFα shedding response. HEK293 cells transfected with an empty vector (Mock), the wild type (WT) or the mutant D_2R-encoding plasmids were subjected to the TGFα shedding assay for their agonist activity to dopamine (grey) or antagonist activity to spiperone (pink) or risperidone (blue) in the presence of 1 μM dopamine. AP-TGFα release response in the absence of any of the compounds was set as a baseline. Data represent mean ± SEM from biologically independent experiments. The numbers of the independent agonist experiments are 11 for WT, 10 for V111A, V115A and I184A, 9 for W90L, L94A, W100A, I183A, T412N and T412A, 8 for V91A, F110A, V91A/V111A and W386L, 5 for F110W, L123W and W90L/F110W, 3 for S121K and S121K/L123W. The numbers of the independent antagonist experiments are 11 for WT, 6 for V111A, V115A and I184A, 5 for W90L, L94A, W100A, I183A, T412N and T412A, 4 for V91A, F110A, F110W, L123W, W386L, W90L/F110W and V91A/V111A.

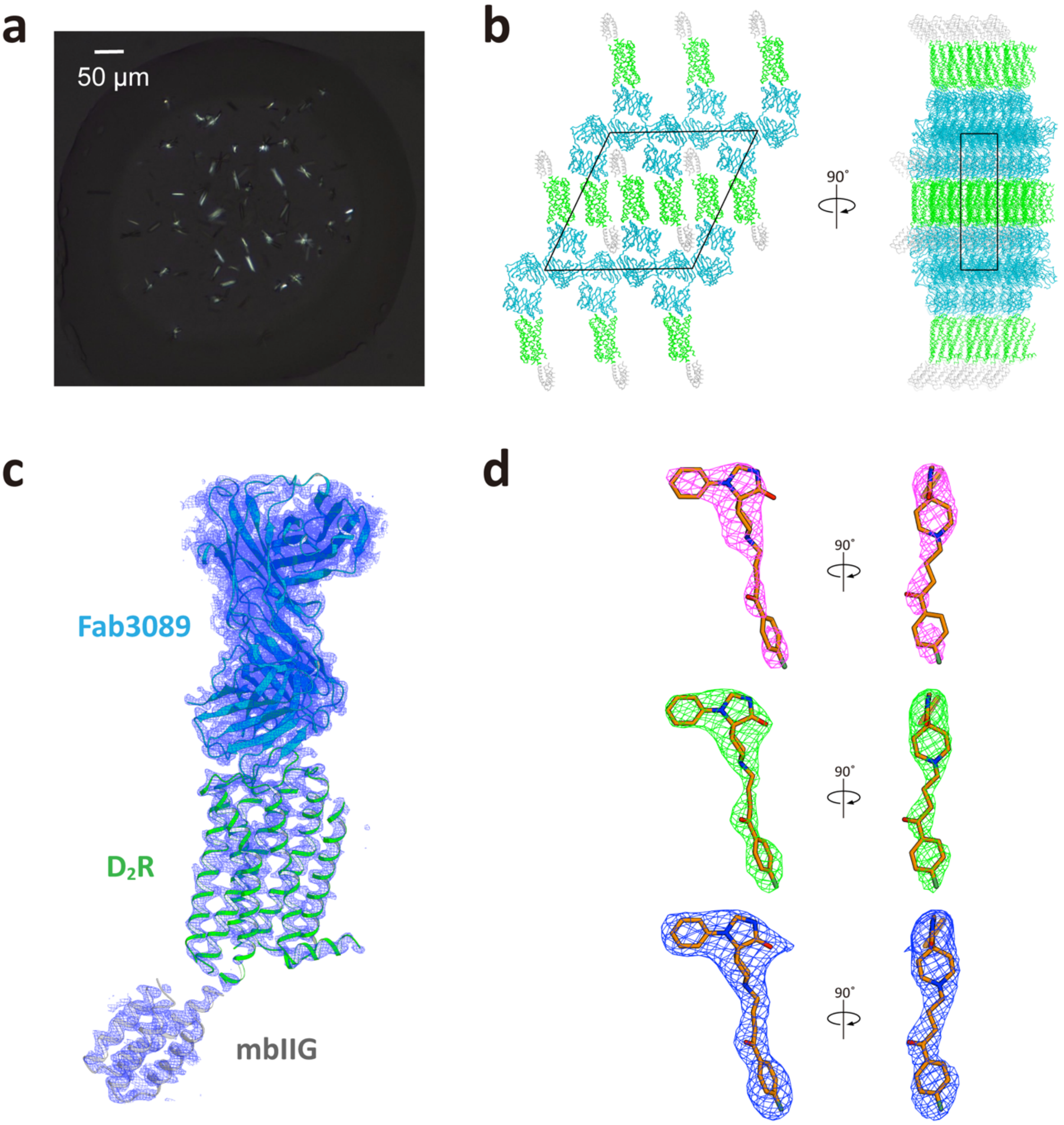

Supplementary Fig. 4. Crystals, crystal packing and density maps of D_2R_{spi}. a, Crystals of D_2R_{spi} under the cross-polarized light. This experiment was repeated independently 5 times with similar results. **b,** Crystal packing of D_2R_{spi}. The unit cell is outlined by the black line. **c,** 2*Fo*-*Fc* electron density map for the D_2R_{spi}-Fab3089 complex contoured at 1.0 σ. **d,** Simulated-annealing composite omit map (upper, in magenta), *Fo*-*Fc* map (middle, in green mesh) and polder map (lower, in blue mesh) of spiperone contoured at 1 σ, 3 σ and 4 σ, respectively.

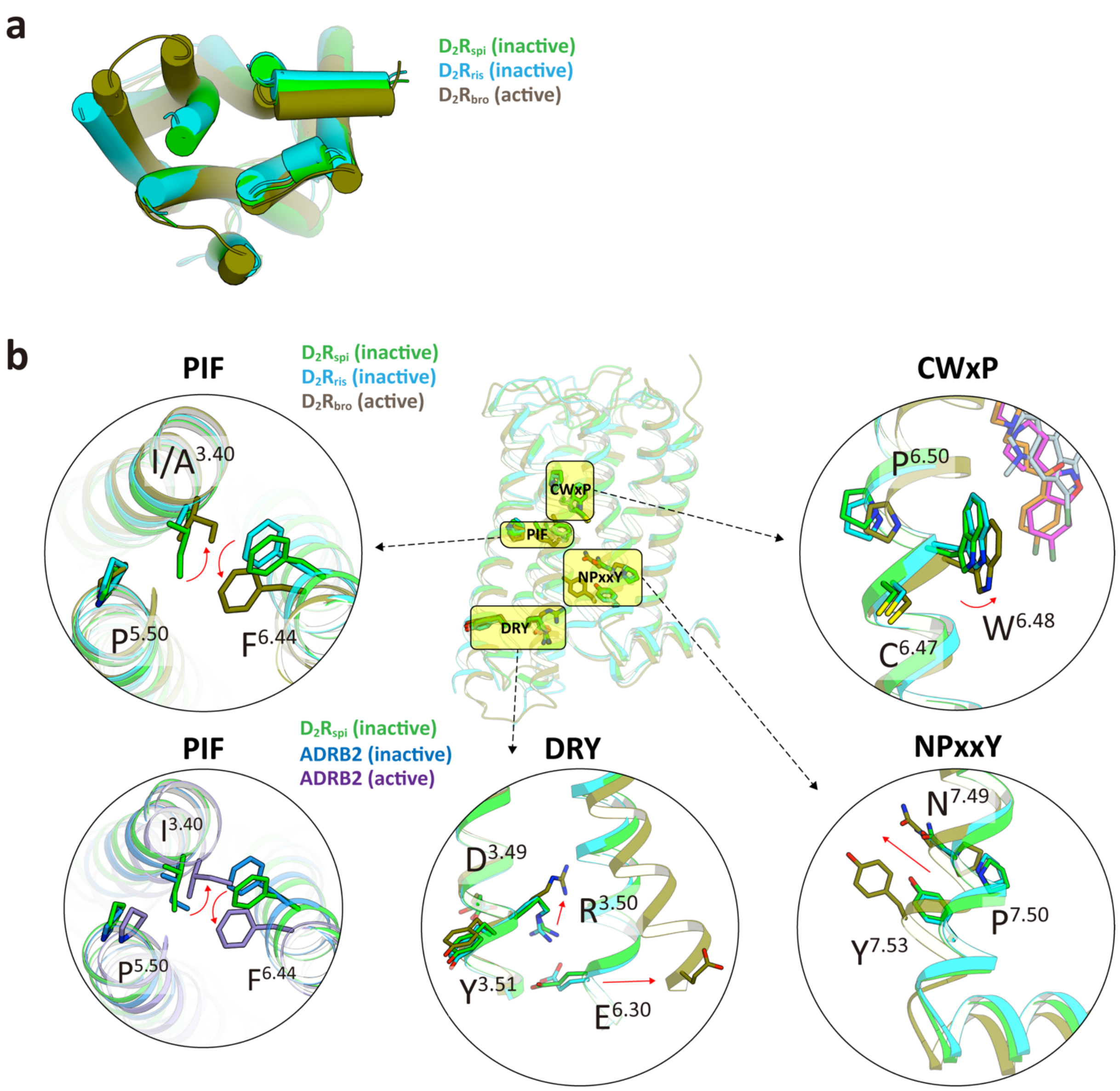

Supplementary Fig. 5. Structural comparison of the seven transmembrane helices and the activation motifs in D_2R and ADRB2. a, Intracellular view of the superposition of D_2R_{spi}, D_2R_{ris} and D_2R_{bro}. Seven transmembrane helices and helix 8 are represented as cylinders. **b,** Superposition of D_2R_{spi}, D_2R_{ris}, D_2R_{bro} around the PIF motif, the CWxP motif, the DRY motif, and the NPxxY motif. The PIF motif of D_2R_{spi} is also compared with those of the inactive state ADRB2 (PDB ID: 5JQH), and the active state ADRB2 (PDB ID: 3SN6). Ligands and side chains are shown as sticks. Red arrows indicate the conformational rearrangements of residues in the activation motifs upon receptor activation. D_2R_{spi} (green), D_2R_{ris} (cyan), D_2R_{bro} (olive), inactive state ADRB2 (blue), active state ADRB2 (purple), spiperone (orange), risperidone (magenta), and bromocriptine (lightblue) are shown.

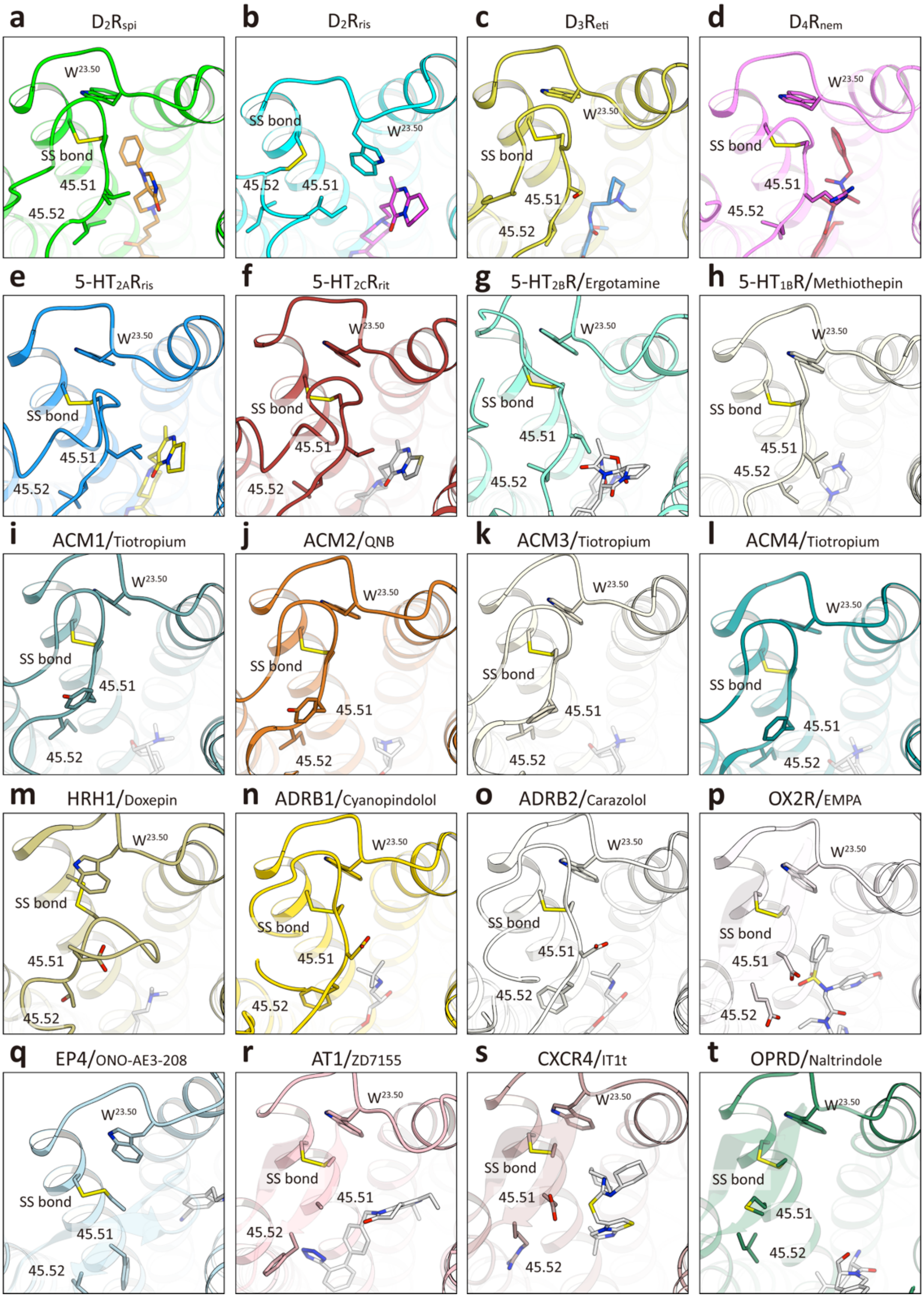

Supplementary Fig. 6. The conserved conformation of Trp$^{23.50}$ on ECL1, the disulfide bridge, and residue$^{45.51}$ and residue$^{45.52}$ on ECL2. Ligands and side chains are shown as sticks. The PDB IDs are shown in parentheses. **a,** D_2R_{spi}. **b,** D_2R_{ris} (6CM4). **c,** D_3R_{eti} (3PBL). **d,** D_4R_{nem} (5WIU). **e,** 5-$HT_{2A}R_{ris}$ (6A93). **f,** 5-$HT_{2C}R_{rit}$ (6BQH). **g,** 5-$HT_{2B}R$ (4IB4). **h,** 5-$HT_{1B}R$ (5V54). **i,** ACM1 (5CXV). **j,** ACM2 (3UON). **k,** ACM3 (4DAJ). **l,** ACM4 (5DSG). **m,** HRH1 (3RZE). **n,** ADRB1 (2RH1). **o,** ADRB2 (2RH1). **p,** OX2R (5WS3). **q,** EP4 (5YWY). **r,** AT1R (4YAY). **s,** CXCR4 (3OE6). **t,** OPRD (4RWA).

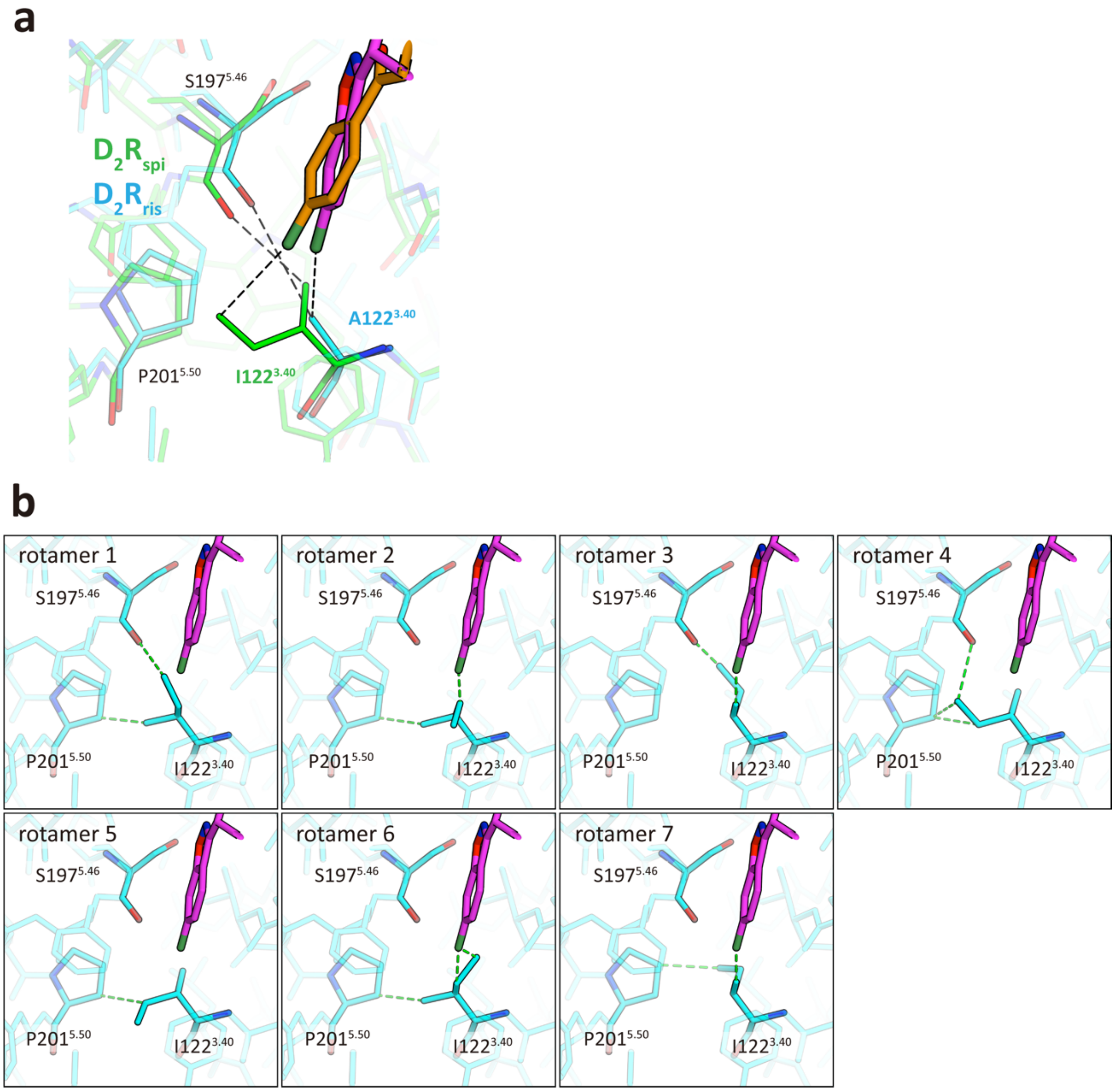

Supplementary Fig. 7. Comparison of D_2R_{spi} with D_2R_{ris} around TM5 and the bottom hydrophobic cleft. a, Close-up view of the superposition of D_2R_{spi} and D_2R_{ris} around I/A122$^{3.40}$. Black dotted lines indicate the contact between I/A122$^{3.40}$ and the ligand or the carbonyl oxygen of S197$^{5.46}$. **b,** The seven allowed side chain rotamers of the I122A$^{3.40}$ mutant of D_2R_{ris} generated using Coot. Green dotted lines indicate the steric contacts between I122$^{3.40}$ and risperidone or residues on TM5. Rotamer 4 represents the inactive conformation of the isoleucine of the PIF motif in the aminergic receptors, including D_2R_{spi}. D_2R_{spi}, D_2R_{ris}, spiperone, and risperidone are indicated in green, cyan, orange, and magenta, respectively.

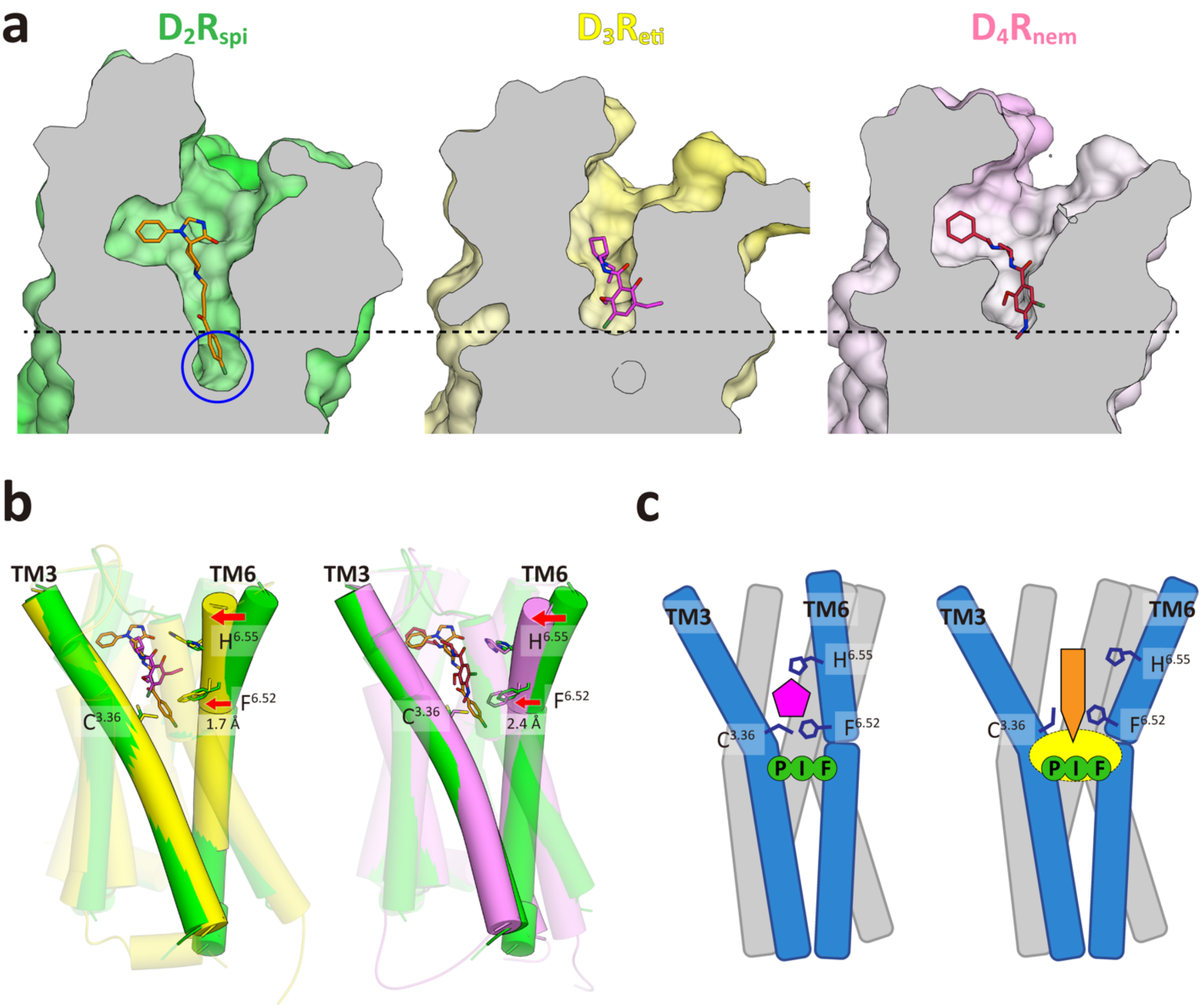

Supplementary Fig. 8. Comparison of the ligand-binding pocket in D_2-class receptors. a, Vertical cross sections of D_2-class receptors. Black dotted line indicates the positions of $C^{3.36}$ and $F^{6.52}$. Blue circle indicates the bottom hydrophobic cleft. **b,** Superposition of the TMs of D_2R_{spi} and D_3R_{eti} (left) or D_4R_{nem} (right). Red arrows indicate the tilt of TM6 to TM3 in D_3R_{eti} and D_4R_{nem} in comparison with D_2R_{spi}. D_2R_{spi}, D_3R_{eti}, D_4R_{nem}, spiperone, eticlopride and nemonapride are shown in green, yellow, pink, orange, magenta and red, respectively. **c,** Schematic representation of two inactive states of D_2-class receptors. Benzamide antipsychotics and butyrophenone or a pyridopyrimidine antipsychotics are shown pink and orange, respectively. The bottom hydrophobic cleft is indicated in yellow. The PIF motif is shown in green.

Supplementary Table 1. RMSD values (Å) among the D_2R structures.

	D_2R_{spi}			D_2R_{ris}			D_2R_{hal}		
	Overall	ΔECL2[1]	7TM[2]	Overall	ΔECL2	7TM	Overall	ΔECL2	7TM
D_2R_{ris}	2.2	1.0	0.8						
D_2R_{hal}	2.1	0.9	0.7	0.9	0.7	0.5			
D_2R_{bro}	2.5	2.5	2.4	3.2	2.6	2.5	3.1	2.4	2.4

[1] Comparison without ECL2.

[2] Comparison of the transmembrane region.

Supplementary Table 2. **Affinities of antipsychotics for mutants and wild-type D_2R**.

	Spiperone Kd ± SEM (nM)	Raclopride Kd ± SEM (nM)	Eticlopride Ki (nM) (pKi± SEM)
Wild type	0.29 ± 0.05	14.0 ± 6.2	0.24 (9.62± 0.04)
Crystallized construct[1]	1.1 ± 0.5		
S121K$^{3.39}$	0.34 ± 0.04	75.6 ± 15.9	1.5 (8.83± 0.13)
L123W$^{3.41}$	0.22 ± 0.06		
S121K$^{3.39}$/L123W$^{3.41}$	0.40 ± 0.07		
I184A	ND[2]		

[1] Expressed in Sf9 cells. Other receptors were expressed in HEK cells.

[2] ND: not determined because of the low expression.

Supplementary Table 3. **Antagonist activities of spiperone against the wild-type (WT) and mutant dopamine 2 receptors.**

	WT (*n* = 16, 10)[1]			W90L$^{2.60}$ (*n* = 9, 5)		
	E_{max} [2]	pEC$_{50}$	EC$_{50}$ [3] (nM)	E_{max}	pEC$_{50}$	EC$_{50}$ (nM)
Dopamine	57.5 ± 1.6	7.91 ± 0.02	16	58.5 ± 1.5	6.78 ± 0.06	170
	pK_B	K_B [3] (pM)	ΔpK_B [4]	pK_B	K_B (pM)	ΔpK_B
Spiperone	11.48 ± 0.11	3.3	0	10.17 ± 0.06	68	-1.42 ± 0.14
Risperidone	9.95 ± 0.08	110	0			
	V91A$^{2.61}$ (*n* = 8, 4)			L94A$^{2.64}$ (*n* = 9, 5)		
	E_{max}	pEC$_{50}$	EC$_{50}$ (nM)	E_{max}	pEC$_{50}$	EC$_{50}$ (nM)
Dopamine	53.8 ± 1.8	8.54 ± 0.03	2.9	55.7 ± 1.1	7.72 ± 0.03	19
	pK_B	K_B (pM)	ΔpK_B	pK_B	K_B (pM)	ΔpK_B
Spiperone	10.62 ± 0.25	24	-0.94 ± 0.15	11.27 ± 0.09	5.4	-0.33 ± 0.11
	W100A$^{23.50}$ (*n* = 9, 5)			F110A$^{3.28}$ (*n* = 8, 4)		
	E_{max}	pEC$_{50}$	EC$_{50}$ (nM)	E_{max}	pEC$_{50}$	EC$_{50}$ (nM)
Dopamine	59.8 ± 1.3	5.95 ± 0.04	1100	56.2 ± 1.4	6.84 ± 0.03	150
	pK_B	K_B (pM)	ΔpK_B	pK_B	K_B (pM)	ΔpK_B
Spiperone	8.71 ± 0.06	2000	-2.95 ± 0.12	10.69 ± 0.09	20	-0.87 ± 0.12
	F110W$^{3.28}$ (*n* = 5, 4)			W90L$^{2.60}$/ F110W$^{3.28}$ (*n* = 5, 4)		
	E_{max}	pEC$_{50}$	EC$_{50}$ (nM)	E_{max}	pEC$_{50}$	EC$_{50}$ (nM)
Dopamine	64.2 ± 3.2	7.11 ± 0.05	77	65.3 ± 3.3	6.56 ± 0.04	270
	pK_B	K_B (pM)	ΔpK_B	pK_B	K_B (pM)	ΔpK_B
Spiperone	10.45 ± 0.08	36	-0.90 ± 0.13	10.13 ± 0.04	75	-1.22 ± 0.17
	V111A$^{3.29}$ (*n* = 10, 6)			V91A$^{2.61}$/V111A$^{3.29}$ (*n* = 8, 4)		
	E_{max}	pEC$_{50}$	EC$_{50}$ (nM)	E_{max}	pEC$_{50}$	EC$_{50}$ (nM)
Dopamine	57.8 ± 1.0	7.69 ± 0.04	20	53.5 ± 1.6	8.81 ± 0.03	1.6
	pK_B	K_B (pM)	pK_B	pK_B	K_B (pM)	ΔpK_B
Spiperone	10.72 ± 0.15	19	-0.83 ± 0.12	10.53 ± 0.17	30	-1.03 ± 0.14
	V115A$^{3.33}$ (*n* = 10, 6)			S121K$^{3.39}$ (*n* = 3, ND)		
	E_{max}	pEC$_{50}$	EC$_{50}$ (nM)	E_{max}	pEC$_{50}$	EC$_{50}$ (nM)
Dopamine	57.2 ± 1.3	6.77 ± 0.02	170	NA[5]	NA	NA
	pK_B	K_B (pM)	ΔpK_B	pK_B	K_B (pM)	ΔpK_B
Spiperone	10.62 ± 0.08	24	-0.93 ± 0.18	ND[6]	ND	ND

	L123W$^{3.41}$ (n = 10, 6)			S121K$^{3.39}$/L123W$^{3.41}$ (n = 3, ND)		
	E_{max}	pEC_{50}	EC_{50} (nM)	E_{max}	pEC_{50}	EC_{50} (nM)
Dopamine	40.4 ± 3.3	9.09 ± 0.04	0.81	NA	NA	NA
	pK_B	K_B (pM)	ΔpK_B	pK_B	K_B (pM)	ΔpK_B
Spiperone	11.71 ±0.13	1.9	0.37 ± 0.16	ND	ND	ND
	I183A$^{45.51}$ (n = 9, 5)			I184A$^{45.52}$ (n = 10, 6)		
	E_{max}	pEC_{50}	EC_{50} (nM)	E_{max}	pEC_{50}	EC_{50} (nM)
Dopamine	55.3 ± 1.0	8.16 ± 0.03	6.9	60.1 ± 1.0	5.93 ± 0.03	1200
	pK_B	K_B (pM)	ΔpK_B	pK_B	K_B (pM)	ΔpK_B
Spiperone	12.35 ±0.11	0.44	0.76 ± 0.11	9.73 ± 0.06	190	-1.83 ± 0.20
Risperidone	10.52 ±0.09	30	0.47 ± 0.11	9.60 ± 0.04	250	-0.44 ± 0.15
	W386L$^{6.48}$ (n = 8, 4)			T412N$^{7.39}$ (n = 9, 5)		
	E_{max}	pEC_{50}	EC_{50} (nM)	E_{max}	pEC_{50}	EC_{50} (nM)
Dopamine	49.6 ± 2.1	5.08 ± 0.03	8300	56.4 ± 1.1	6.73 ± 0.03	180
	pK_B	K_B (pM)	ΔpK_B	pK_B	K_B (pM)	ΔpK_B
Spiperone	NA	NA	NA	10.01 ± 0.05	98	-1.59 ± 0.15
	T412A$^{7.39}$ (n = 9, 5)					
	E_{max}	pEC_{50}	EC_{50} (nM)			
Dopamine	56.5 ± 1.5	6.74 ± 0.04	180			
	pK_B	K_B (pM)	ΔpK_B			
Spiperone	10.15 ±0.10	71	-1.45 ± 0.19			

Data represent mean ± SEM of the indicated numbers of independent experiments.

1 (n = 16, 10) indicates that the experiments were repeated 16 and 10 times to determine the pEC_{50} and pK_B values, respectively.

2 E_{max}: %AP-TGFα release.

3 EC_{50} and K_B were calculated from the mean pEC_{50} and pK_B values, respectively.

4 $\Delta pK_B = pK_{B(mutant)} - pK_{B(WT)}$, which was calculated for each experiment performed in parallel.

5 NA: no detectable activity.

6 ND: not determined because of lack of detectable dopamine response.

Supplementary Table 4. Residues within 4.5 Å from spiperone in D_2R_{spi} and their equivalents in the related aminergic receptors.

D_2R	D_3R	D_4R	5-$HT_{2A}R$	5-$HT_{2C}R$
$W90^{2.60}$	W85	L90	V130	L109
$V91^{2.61}$	V86	F91	S131	S110
$L94^{2.64}$	L89	S94	T134	A113
$F110^{3.28}$	F106	L111	W151	W130
$V111^{3.29}$	V107	M112	I152	I131
$C182^{45.50}$	C181	C185	C227	C207
$I183^{45.51}$	S182	R186	L228	V208
$I184^{45.52}$	I183	L187	L229	L209
$D114^{3.32}$	D110	D115	D155	D134
$T412^{7.39}$	T369	T434	V366	V354
$Y416^{7.43}$	Y373	Y438	Y370	Y358
$V115^{3.33}$	V111	V116	V156	V135
$F389^{6.51}$	F345	F410	F339	F327
$C118^{3.36}$	C114	C119	S159	S138
$T119^{3.37}$	T115	T120	T160	T139
$I122^{3.40}$	I118	I123	I163	I142
$S197^{5.46}$	S196	S200	S242	A222
$F198^{5.47}$	F197	F201	F243	F223
$F382^{6.44}$	F338	F403	F332	F320
$W386^{6.48}$	W342	W407	W336	W324
$F390^{6.52}$	F346	F411	F340	F328

Supplementary Table 5. Primers for site-directed mutagenesis

Oligonucleotides primer	Forward	Reverse
D_2R_W90L	ATGCCCCTGGTTGTCTACCTGGAGGTG	GACAACCAGGGGCATGACCAGTGTGGC
D_2R_V91A	CCCTGGGCCGTCTACCTGGAGGTGGTA	GTAGACGGCCCAGGGCATGACCAGTGT
D_2R_L94A	GTCTACGCCGAGGTGGTAGGTGAGTGG	CACCTCGGCGTAGACAACCCAGGGCAT
D_2R_W100A	GGTGAGGCCAAATTCAGCAGGATTCAC	GAATTTGGCCTCACCTACCACCTCCAG
D_2R_F110A	GACATCGCCGTCACTCTGGACGTCATG	AGTGACGGCGATGTCACAGTGAATCCT
D_2R_F110W	GACATCTGGGTCACTCTGGACGTCATGATGTGC	AGTGACCCAGATGTCACAGTGAATCCTGCTGAA
D_2R_V111A	ATCTTCGCCACTCTGGACGTCATGATG	CAGAGTGGCGAAGATGTCACAGTGAAT
D_2R_V115A	CTGGACGCCATGATGTGCACGGCGAGC	CATCATGGCGTCCAGAGTGACGAAGAT
D_2R_S121K	ACGGCGAAGATCCTGAACTTGTGTGCCATCAGC	CAGGATCTTCGCCGTGCACATCATGACGTCCAG
D_2R_L123W	AGCATCTGGAACTTGTGTGCCATCAGCATCGAC	CAAGTTCCAGATGCTCGCCGTGCACATCATGAC
D_2R_S121K/L123W	ACGGCGAAGATCTGGAACTTGTGTGCCATCAGCATCGAC	CAAGTTCCAGATCTTCGCCGTGCACATCATGACGTCCAG
D_2R_I183A	GAGTGCGCCATTGCCAACCCGGCCTTC	GGCAATGGCGCACTCGTTCTGGTCTGC
D_2R_I184A	TGCATCGCCGCCAACCCGGCCTTCGTG	GTTGGCGGCGATGCACTCGTTCTGGTC
D_2R_W386L	ATCTGCCTGCTGCCCTTCTTCATCACA	GGGCAGCAGGCAGATGATGAACACGCC
D_2R_T412N	GCCTTCAACTGGCTGGGCTATGTCAAC	CAGCCAGTTGAAGGCGCTGTACAGGAC
D_2R_T412A	GCCTTCGCCTGGCTGGGCTATGTCAAC	CAGCCAGGCGAAGGCGCTGTACAGGAC
