# Supplementary material for: Structure of the dopamine D2 receptor in complex with the antipsychotic drug spiperone
Source: Nat Commun. 2020 Dec 22;11:6442. doi: 10.1038/s41467-020-20221-0 (PMC7755896; doi:10.1038/s41467-020-20221-0)
Supplement: Supplementary file 3 — Reporting Summary [file 41467_2020_20221_MOESM3_ESM.pdf]

nature research

Corresponding author(s): So Iwata, Tatsuro Shimamura

Last updated by author(s): Nov 10, 2020

Reporting Summary

Nature Research wishes to improve the reproducibility of the work that we publish. This form provides structure for consistency and transparency in reporting. For further information on Nature Research policies, see Authors & Referees and the Editorial Policy Checklist .

Statistics

For all statistical analyses, confirm that the following items are present in the figure legend, table legend, main text, or Methods section.

n/a	Confirmed	
☐	☒	The exact sample size (*n*) for each experimental group/condition, given as a discrete number and unit of measurement
☐	☒	A statement on whether measurements were taken from distinct samples or whether the same sample was measured repeatedly
☒	☐	The statistical test(s) used AND whether they are one- or two-sided<br>*Only common tests should be described solely by name; describe more complex techniques in the Methods section.*
☒	☐	A description of all covariates tested
☒	☐	A description of any assumptions or corrections, such as tests of normality and adjustment for multiple comparisons
☐	☒	A full description of the statistical parameters including central tendency (e.g. means) or other basic estimates (e.g. regression coefficient) AND variation (e.g. standard deviation) or associated estimates of uncertainty (e.g. confidence intervals)
☒	☐	For null hypothesis testing, the test statistic (e.g. *F*, *t*, *r*) with confidence intervals, effect sizes, degrees of freedom and *P* value noted<br>*Give P values as exact values whenever suitable.*
☒	☐	For Bayesian analysis, information on the choice of priors and Markov chain Monte Carlo settings
☒	☐	For hierarchical and complex designs, identification of the appropriate level for tests and full reporting of outcomes
☒	☐	Estimates of effect sizes (e.g. Cohen's *d*, Pearson's *r*), indicating how they were calculated

Our web collection on statistics for biologists contains articles on many of the points above.

Software and code

Policy information about availability of computer code

Data collection	Cheetah
Data analysis	CrystFEL 0.8.0, XGANDALF, Phaser, Phenix 1.16, Coot 0.8.9, CueMol 2.2.3, PyMol 2.3.0, MolProbity

For manuscripts utilizing custom algorithms or software that are central to the research but not yet described in published literature, software must be made available to editors/reviewers. We strongly encourage code deposition in a community repository (e.g. GitHub). See the Nature Research guidelines for submitting code & software for further information.

Data

Policy information about availability of data

All manuscripts must include a data availability statement. This statement should provide the following information, where applicable:

- Accession codes, unique identifiers, or web links for publicly available datasets
- A list of figures that have associated raw data
- A description of any restrictions on data availability

The coding sequence of human D2R is available in UniProt with accession code P14416 [https://www.uniprot.org/uniprot/P14416]. The protein coordinate and atomic structure factor have been deposited in the Protein Data Bank (PDB) with accession code 7DFP [http://doi.org/10.2210/pdb7DFP/pdb]. The raw diffraction images have been deposited to CXIDB (https://cxidb.org/) with accession code 110. Other data are available from the corresponding authors upon reasonable request. Source data are provided with this paper.

Field-specific reporting

Please select the one below that is the best fit for your research. If you are not sure, read the appropriate sections before making your selection.

- [x] Life sciences
- [ ] Behavioural & social sciences
- [ ] Ecological, evolutionary & environmental sciences

For a reference copy of the document with all sections, see nature.com/documents/nr-reporting-summary-flat.pdf

Life sciences study design

All studies must disclose on these points even when the disclosure is negative.

Sample size	The sample size was determined based on the previous studies (refs 51, 52, 56).
Data exclusions	No data was excluded from the analysis.
Replication	All activity measurements were performed repeatedly with the statements of the sample size in the text and methods section.
Randomization	There is no group allocation in the study, so no blinding is needed.
Blinding	There is no group allocation in the study, so no randomization is needed.

Reporting for specific materials, systems and methods

We require information from authors about some types of materials, experimental systems and methods used in many studies. Here, indicate whether each material, system or method listed is relevant to your study. If you are not sure if a list item applies to your research, read the appropriate section before selecting a response.

Materials & experimental systems

n/a	Involved in the study	
	x	Antibodies
	x	Eukaryotic cell lines
x		Palaeontology
	x	Animals and other organisms
x		Human research participants
x		Clinical data

Methods

n/a	Involved in the study	
x		ChIP-seq
x		Flow cytometry
x		MRI-based neuroimaging

Antibodies

Antibodies used	Fab3089
Validation	The Fab fragment antibody (Fab3089) specific for the dopamine D2 receptor is our original as described in the manuscript.

Eukaryotic cell lines

Policy information about cell lines

Cell line source(s)	Sf9 and HEK293 cells were purchased from Thermo Fisher Scientific.
Authentication	Non of cell lines were authenticated.
Mycoplasma contamination	The cell lines were not tested for Mycoplasma contamination.
Commonly misidentified lines (See ICLAC register)	None of commonly misidentified lines were used in this study.

Animals and other organisms

Policy information about studies involving animals; ARRIVE guidelines recommended for reporting animal research

Laboratory animals	MRL/lpr mice

Wild animals	Not applicable
Field-collected samples	Not applicable
Ethics oversight	All animal experiment described in this study conformed to the guidelines outlined in the Guide for the Care and Use of Laboratory Animals of Japan and were approved by Kyoto University Animal Care Committee (Approval No. MedKyo 16043)

Note that full information on the approval of the study protocol must also be provided in the manuscript.
